# HIGH-FLOW NASAL CANNULA POST-TRACHEAL EXTUBATION IN A CHILD WITH UPPER AIRWAY OBSTRUCTION: CASE REPORT

**DOI:** 10.1590/1984-0462/;2018;36;3;00010

**Published:** 2018

**Authors:** José Colleti, Tâmara Eleamen Longui, Werther Brunow de Carvalho

**Affiliations:** a Hospital Santa Catarina, São Paulo, Brasil.; b Hospital Municipal Menino Jesus, São Paulo, Brasil.; c Universidade de São Paulo, São Paulo, Brasil.

**Keywords:** Acute laryngitis, High flow nasal cannula, Tracheal extubation, Laringite aguda, Cânula nasal de alto fluxo, Extubação traqueal

## Abstract

**Objective:**

To report a case of a patient who required tracheal intubation in a pediatric emergency department due to acute laryngitis and that, after the planned extubation, has successfully used the high-flow nasal cannula, which possibly prevented extubation failure.

**Case description:**

A male 8-month-old child was admitted to the pediatric emergency room with acute respiratory distress due to a high airway obstruction secondary to severe acute laryngitis. He was immediately intubated and referred to the pediatric intensive care unit. He presented extubation failure due to a significant laryngeal edema evidenced by bronchoscopy. In the second attempt to extubate, he presented respiratory distress, but, after the use of the high-flow nasal cannula, he became stable, reducing the heart and respiratory frequencies, and the extubation was successful.

**Comments:**

The use of the high-flow nasal cannula was effective and presented good response in this patient with acute laryngitis, suggesting that it is a possible adjuvant for the treatment, avoiding worsening respiratory conditions and the need for reintubation.

## RESUMO

**Objetivo:**

Relatar o caso de um lactente que necessitou de intubação traqueal no setor de emergência pediátrica por conta de laringite aguda grave e que, após a extubação traqueal programada, fez uso, com sucesso, da cânula nasal de alto fluxo, a qual, possivelmente, evitou a falha da extubação traqueal.

**Descrição do caso:**

Paciente masculino, 8 meses de idade, admitido no pronto atendimento de pediatria com desconforto respiratório agudo por causa de obstrução alta de vias aéreas secundária à laringite aguda grave. Foi imediatamente intubado e encaminhado para a unidade de terapia intensiva (UTI) pediátrica. Apresentou falha de extubação em função de edema importante de laringe evidenciado por broncoscopia, o que contraindicou a extubação. Na segunda tentativa de extubação, o paciente apresentou desconforto respiratório, melhorando após uso da cânula nasal de alto fluxo, com redução da frequência cardíaca e respiratória, possibilitando a extubação com sucesso.

**Comentários:**

O uso da cânula nasal de alto fluxo foi eficaz e apresentou boa resposta nesse paciente com laringite aguda, sugerindo ser um possível adjuvante para o tratamento, evitando-se a piora do quadro respiratório e da necessidade de reintubação.

## INTRODUCTION

Acute laryngitis, in general, is caused by the viral infection of upper airways. It mostly affects children aged from 6 months to 3 years, and its peak incidence age is 2 years, especially among male infants.^1^ The most common etiological agents are the parainfluenza virus, influenza, metapneumovirus, adenovirus, coronavirus and the respiratory syncytial virus.^1^ ^,^ ^2^ The infection begins at the nasopharynx and spreads along the respiratory epithelium, causing inflammation of the subglottic portion of the larynx. Congestion and edema of this region lead to a variable degree of airway obstruction, restricting the air flow entry, causing respiratory difficulties.^1^ ^,^ ^2^ ^,^ ^3^ In 24 to 48 hours, the situation is aggravated with mild to severe obstruction, presence of barking cough, dysphonia, aphonia, or hoarse cry, and inspiratory stridor. In extreme cases, besides intensive dyspnea and agitation, there is paleness, cyanosis, numbness, seizures, apnea, and death.^2^

The high-flow nasal cannula is an alternative modality of medicinal gas administration, with heated and humidified oxygen in flows that range, in pediatrics, from 1 to 2 L/kg, and which has been used for ventilatory support in several clinical conditions.^4^ ^,^ ^5^ ^,^ ^6^ ^,^ ^7^ ^,^ ^8^ ^,^ ^9^

We reported the case of a patient, in the pediatric emergency room, who needed tracheal intubation due to severe acute laryngitis, and who, after the scheduled tracheal extubation, successfully used the high-flow nasal cannula, which possibility prevented the need for a new tracheal intubation.

## CASE DESCRIPTION

**Chart 1:**
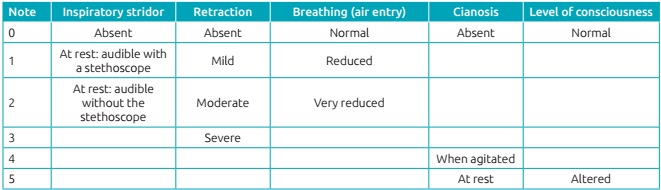
Westley’s scale for the clinical evaluation of acute laryngitis.

Male, 8-month-old patient, weighting 11 kg, living in the city of São Paulo (São Paulo), was hospitalized in the pediatric emergency room presenting with laryngeal inspiratory stridor associated with suprasternal notch and subdiaphragmatic retraction, classified as severe (score 11) in the Westley’s scale (Chart 1). He evolved to severe acute respiratory failure, presenting respiratory frequency (RF) of 70 breaths per minute (bpm) and heart rate (HR) higher than 200 beats per minute (bpm), requiring tracheal intubation and being referred to the pediatric intensive care unit (ICU).

Classification: mild (<3); moderate (3 to 5); severe (>6).

As personal history, the child was born in a C-section, at term, and was intubated for 17 days in the pediatric ICU due to early respiratory distress. He presented with tracheal extubation failure caused by high respiratory distress in that hospitalization, evolving without intercurrences after that period.

**Figure 1:**
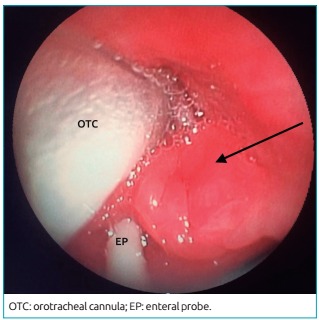
Bronchoscopy image showing mucosal edema (arrow) involving the orotracheal cannula and the enteral probe.

In the pediatric ICU, dexamethasone (0.6 mg/kg/day) was introduced on the first day of hospitalization, and the patient remained in mechanical ventilation, requiring ventilator support: fraction of inspired oxygen (FiO_2_): 0.25; inspiratory time: 0.79 seconds; RF: 30bpm; positive end expiratory pressure: 5 cmH_2_O; pressure support: 14 cmH_2_O; pressure control: 16 cmH_2_O; and current volume: 85 mL. He remained intubated, on midazolam (0.2 mg/kg/h) and fentanyl (2 mcg/kg/h), without the need for vasoactive medication, remaining clinically stable. Six days after hospitalization, flexible bronchoscopy was carried out to assess the conditions of the mucosa before indicating extubation, since the patient had been intubated for six days without signs of tracheal peri-cannula ventilator escape. Bronchoscopy revealed “major mucosal edema, involving the intratracheal cannula” (Figure 1), so the choice was to maintain the tracheal cannula, without progression of bronchoscopy through the trachea. The infant was intubated, stable, on dexamethasone and medication for sedation and analgesia. Seven days after bronchoscopy, he demonstrated signs of escape through the tracheal cannula, shown by auscultation and observed in the monitor of the ventilation equipment (Servo-i^®^, Maquet, Rastatt, Germany). A new flexible bronchoscopy was carried out for the supervised extubation. Bronchoscopy showed “larynx with discrete posterior wall edema, with good mobilization of vocal folds and air escape between the tracheal cannula and the larynx”.

**Figure 2:**
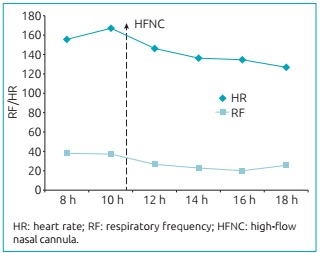
Reduction of heart rate and respiratory frequency after the use of high-flow nasal cannula (dotted arrow).

For the programming of the tracheal extubation, a thoracic X-ray was performed and did not show any abnormalities nor arterial blood gas (pH: 7.50; pO_2_: 120 mmHg; pCO_2_: 32 mmHg; sodium bicarbonate: 21 mEq/L; base excess: +1; SatO_2_: 98%). Tracheal extubation was conducted and the use of nasal oxygen catheter began, with 2 L/minute, presenting with mild laryngeal stridor, with improvements after inhalation with one ampoule of budesonide (0.25 mg). Forty-eight hours after the extubation, the patient evolved to severe acute respiratory failure, with major laryngeal stridor, vacillation of the wings of the nose, and increased HR until 167 bmp, and RF of 39 bpm, indicating a new tracheal intubation. The choice was to start support with high-flow nasal cannula (Optiflow Junior^®^, Fisher & Paykel, Auckland, New Zealand) at 15 L/minute, FiO2: 25%, with significant improvement in the respiratory situation, as well as reduced HR and RF (Figure 2). The infant was comfortable in the high-flow nasal cannula, preventing tracheal reintubation.

The patient remained with the high-flow nasal cannula for nine days, and showed improvements in high respiratory discomfort. There was a transition from the high-flow nasal cannula to nebulization with oxygen at 2 L/minute for one more day, being discharged to pediatric nursery in good clinical conditions.

Before discharge, since the patient presented with persistent laryngeal stridor, and because of the previous history of intubation for 17 days, and extubation failure in the neonatal period, a new flexible bronchoscopy was performed to investigate the after-effects in the airways, showing “hyperemia of the larynx and subglottic region with edema and granular surface”.

## DISCUSSION

In our report, the patient presented with severe acute laryngitis and benefitted from the use of high-flow nasal cannula in the tracheal post-extubation period, due to signs of persistent airway obstruction after 15 days of tracheal intubation, preventing possible after-effects of tracheal reintubation.

The obstruction of airways is one of the most common problems in pediatric medical services, and leads to significant morbidity.^3^ In acute laryngitis, diagnosis is clinical, and the treatment ranges according to the level of severity. The Westley’s scale is used to classify severity.^2^ This patient was classified with 11 points, considered severe, and received the necessary care in the pediatric emergency sector.

It is important to mention that the infant was submitted to tracheal intubation in the emergency room, which, associated with the prolonged time of tracheal intubation in the neonatal period, may have contributed with the persistent glottis edema, and the difficulty in tracheal extubation. It is important to consider the possibility that the patient has a latent problem coming from the neonatal period, result of a previous tracheal intubation, since, in acute laryngitis, a review by Gelbart et al. showed that the mean time of tracheal intubation is 60 hours.^10^

When tracheal intubation takes place in the emergency sector, it is important that the most experienced physician at the location performs the tracheal intubation, since an edema associated with agitation may make the procedure more difficult. In situations in which there is no success in tracheal intubation, the edema may be aggravated, preventing new attempts. In these cases, it is essential that the medical service has a protocol for airway access involving the anesthesia and/or ear, nose and throat teams, which contemplates the performance of urgent tracheostomy or cricothyroidotomy.^3^ ^,^ ^11^ ^,^ ^12^ ^,^ ^13^

The high-flow nasal cannula presents an action mechanism which, even if not completely clear, has been addressed at improving gas exchange for purifying the dead space and causing positive pharyngeal pressure, which can, up to a certain point, be transmitted to the distal airways, providing low level of positive expiratory pressure.^4^ ^,^ ^5^ ^,^ ^9^

In the case presented, the patient used the high-flow nasal cannula and obtained benefits from the improvement in the respiratory pattern, which possibly prevented tracheal reintubation and the complications from it. The use of the high-flow nasal cannula was efficient and pointed to a good response in acute laryngitis, suggesting it is a possible adjuvant for treatment, avoiding the worsening of the respiratory situation and the need for reintubation. There are studies in adults comparing the use of the high-flow nasal cannula versus non-invasive ventilation in tracheal extubation, and they show that the high-flow nasal cannula is not inferior to noninvasive ventilation; however, these studies do not specifically address acute laryngitis.^14^ In the medical literature, we do not know of medical reports regarding the use of high-flow nasal cannula for this purpose.

Further investigations are necessary in patients with upper airway obstruction to assess the use of non-invasive ventilation in the tracheal post-extubation period.
